# Thrombotic microangiopathy associated with calcineurin inhibitor induction doses in patients with a kidney transplant

**DOI:** 10.1002/ccr3.9125

**Published:** 2024-06-28

**Authors:** Alfredo Gutiérrez‐Govea, Basilio Jalomo‐Martínez, Luis Alberto Evangelista‐Carrillo, José Ignacio Cerrillos‐Gutiérrez, Miguel Medina‐Pérez

**Affiliations:** ^1^ Department of Nephrology and Organ Transplant Unit Specialties Hospital, National Western Medical Centre, Mexican Social Security Institute Guadalajara Jalisco Mexico; ^2^ University Health Sciences Center University of Guadalajara Guadalajara Jalisco Mexico

**Keywords:** calcineurin inhibitor, induction, kidney transplant, nephrology, thrombotic microangiopathy

## Abstract

We present a case of a 23‐year‐old male who developed thrombotic microangiopathy associated with the induction dose of tacrolimus. Get an early diagnosis and give timely treatment of thrombotic microangiopathy is essential to improve the prognosis of the kidney transplant.

## Graphical abstract

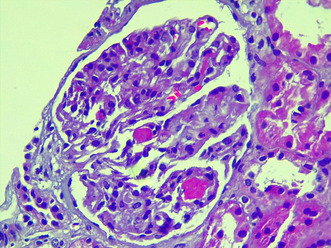

## 1 INTRODUCTION

Thrombotic microangiopathy (TMA) is a rare complication (0.8%–14% of transplant patients), a severe complication frequently associated with graft dysfunction.^1^ TMA can occur de novo and is frequently related to immunosuppressive therapy, humoral rejection, or a history of hemolytic uremic syndrome.^1^, ^2^ In drug‐induced TMA (DITMA), the mechanism of endothelial damage induced by different drugs is heterogeneous and not completely understood; calcineurin inhibitors can cause TMA with a cumulative effect dose that mainly affects kidney endothelial cells on most occasions. It is a potentially life‐threatening disease if it is not resolved in time.^3^ There are only reports of cases of TMA associated with calcineurin inhibitor induction doses during kidney transplantation.^2^

## 2 CLINICAL CASE

**FIGURE 1:**
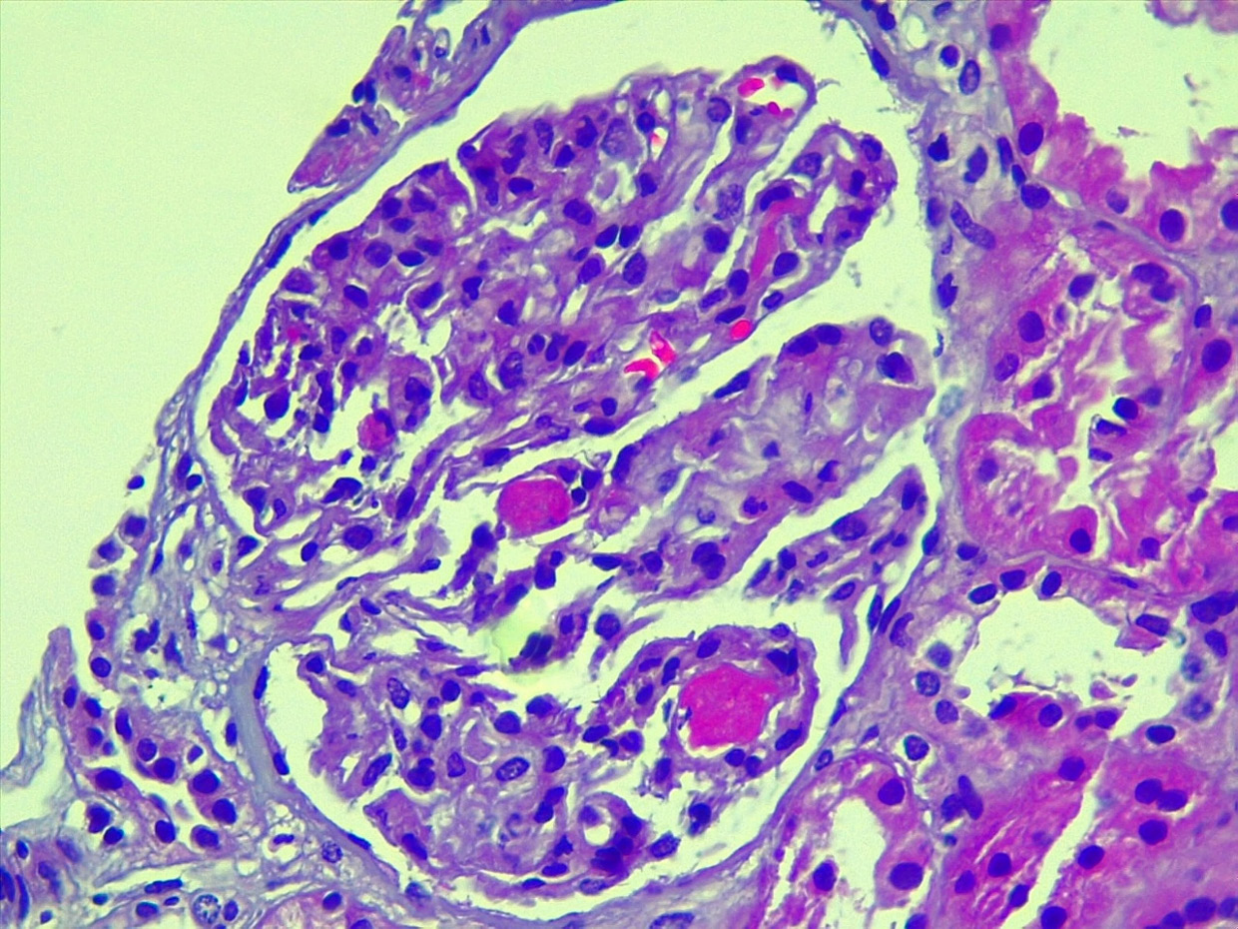
H&E stain 40×: Fibrin microthrombi in glomerular capillary lumens.

**FIGURE 2:**
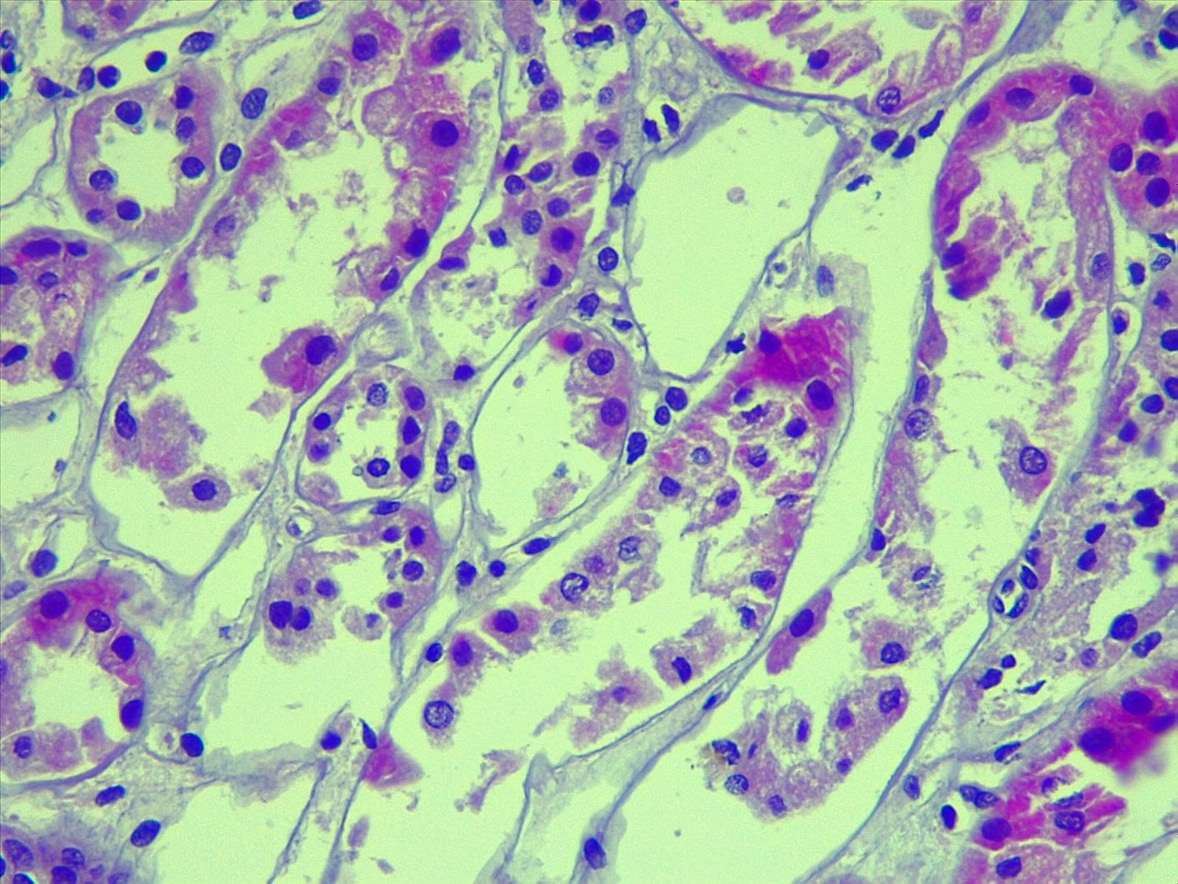
H&E stain 40×: Detachment of the tubular epithelium into the tubular lumens.

**FIGURE 3:**
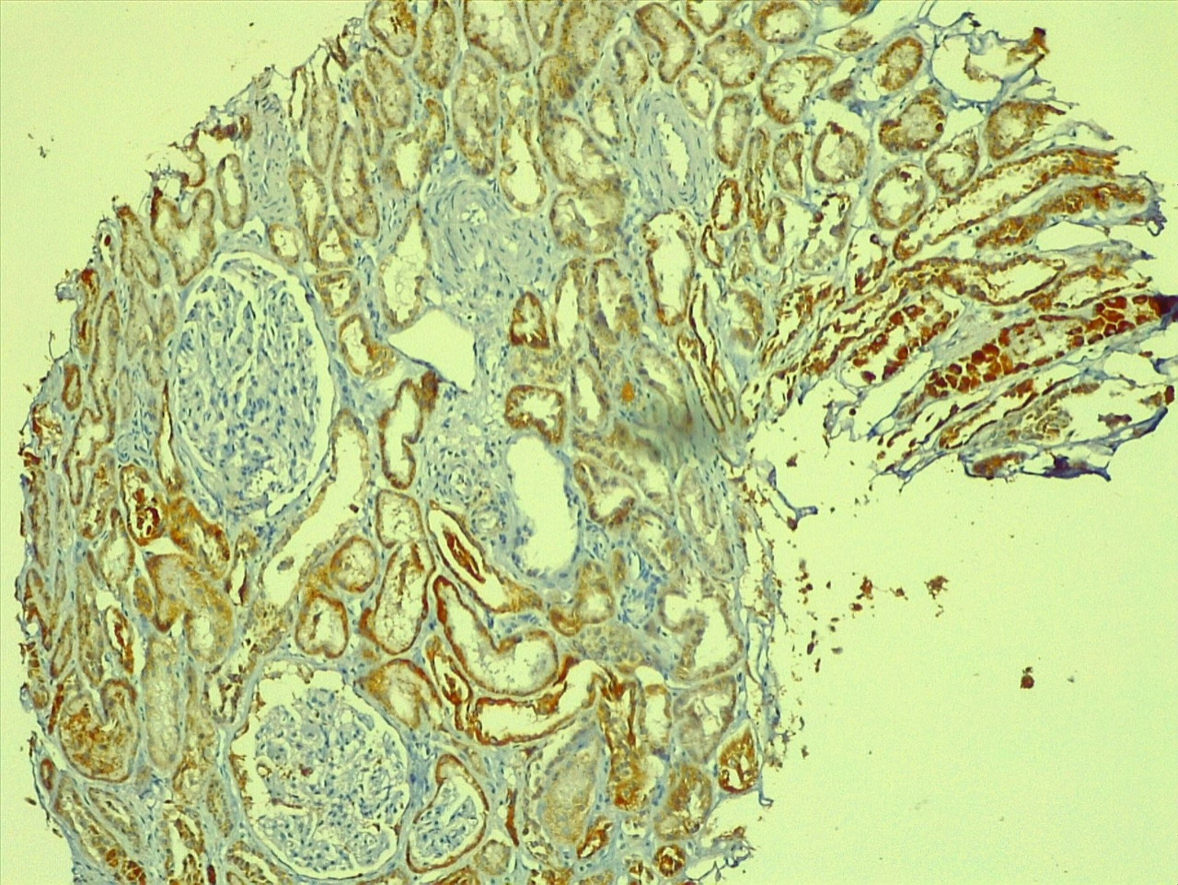
C4d indirect immunoperoxidase: Negative in peritubular and glomerular capillaries.

A 23‐year‐old man diagnosed with 12‐year‐old CKD (chronic kidney disease) for focal segmental glomerulosclerosis was in replacement therapy peritoneal dialysis modality for 3 years. Kidney transplantation was performed in 2021 from a living‐related donor (48‐year‐old mother), same blood group, shared 1 HLA (human leukocyte antigens) haplotype, and cross‐matched negative by flow cytometry. The induction was done with Basiliximab, tacrolimus (0.12 mg/kg/day), mycophenolic acid, and methylprednisolone 500 mg. During surgery, he had a left renal graft, one artery, and one vein, warm ischemia 1 min 8 s, cold ischemia 58 min, a double J catheter was not required, and no apparent complications. However, albuminuria (393 mg/g) was detected 4 days after kidney transplantation. A renal graft biopsy was performed, reporting 21 glomeruli by optical microscope, 4 of them with microthrombi (Figure 1) and data of acute tubular necrosis (Figure 2), C4d negative (Figure 3). It has been made peripheral blood smear without evidence of hemolysis, DSA (donor‐specific antibodies) was negative, and a PLASMIC score of 2 points. He presented elevated urea and decreased urinary volumes, requiring hemodialysis therapy. Tacrolimus was suspended, and a change to sirolimus was made with a significant decrease in urea and creatinine.

## 3 DISCUSSION

De novo TMA is more common in transplant patients, with an incidence of approximately 0.8%.^2^ Other studies report a 5%–15% incidence, ending in graft loss in 45%.^2^, ^3^ It can be triggered by renal ischemia, antibody‐mediated rejection, neoplasms, viral infections (parvovirus B19, BK virus, HIV, etc.), antibodies, anti‐phospholipids, and the use of some drugs (tacrolimus 1%).^2^ The mechanism of de novo TMA secondary to calcineurin inhibitor is not precise. It is believed to be due to its vasoconstrictor effect, endothelial toxicity, and prothrombotic effect.^2^ Approximately 30% of transplant patients do not present the classical TMA features with hemolysis and/or thrombocytopenia.^3^ The reported cases have not shown an association with serum levels of calcineurin inhibitor, and the treatment is to decrease the dose of immunosuppressive therapy or change to another immunosuppressant.^2^ Calcineurin inhibitor induction doses may be associated with thrombotic microangiopathy. Therefore, diagnosis and treatment early have a critical role in renal graft prognosis.

## AUTHOR CONTRIBUTIONS

**Alfredo Gutiérrez‐Govea:** Project administration; writing – original draft. **Basilio Jalomo‐Martínez:** Supervision. **Luis Alberto Evangelista‐Carrillo:** Conceptualization; writing – review and editing. **José Ignacio Cerrillos‐Gutiérrez:** Validation. **Miguel Medina‐Pérez:** Formal analysis; investigation.

## FUNDING INFORMATION

No external funding was received.

## CONFLICT OF INTEREST STATEMENT

The authors had no conflict of interest to disclosure.

## ETHICS STATEMENT

Written informed consent was obtained from the patient to publish this report in accordance with de journal's patient consent policy.

## Data Availability

All basic clinical data have been reported. Physiological assessment data for all treatments and diagnoses are available upon request from the corresponding author.
